# Treatment of Chronic Neck Pain in Patients with Forward Head Posture: A Systematic Narrative Review

**DOI:** 10.3390/healthcare11192604

**Published:** 2023-09-22

**Authors:** Seoyon Yang, Mathieu Boudier-Revéret, You Gyoung Yi, Kee Yong Hong, Min Cheol Chang

**Affiliations:** 1 Department of Rehabilitation Medicine, School of Medicine, Ewha Woman’s University Seoul Hospital, Seoul 07804, Republic of Korea; seoyonyang@gmail.com (S.Y.); lyk861124@gmail.com (Y.G.Y.); 2 Department of Physical Medicine and Rehabilitation, Centre Hospitalier de l’Université de Montréal, Montreal, QC H2X 0A9, Canada; mathieu.boudier.reveret@umontreal.ca; 3 Cheonho S Orthopedic Clinic, 1015, Cheonho-daero, Gangdong-du, Seoul 06014, Republic of Korea; emilio13@naver.com; 4 Department of Physical Medicine and Rehabilitation, College of Medicine, Yeungnam University, Daegu 42415, Republic of Korea

**Keywords:** neck pain, chronic neck pain, forward head posture, treatment

## Abstract

(1) Background: Forward head posture (FHP) is one of the most common cervical postural deviations and is characterized by head protrusion or forward head placement in relation to the shoulder in the sagittal plane. Patients with FHP often experience neck pain and disability. The aim of this study was to investigate whether treatment programs are effective in the management of neck pain in patients with FHP. (2) Methods: A MEDLINE (PubMed), Embase, Cochrane Library, and Scopus database search was conducted for English language articles on patients with chronic neck pain and FHP published until 12 April 2023. To identify potentially relevant articles, the following key search phrases were combined: ‘forward head posture’ and ‘pain’. After searching, 2516 potentially relevant articles were identified. After reading the titles and abstracts and assessing their eligibility based on full-text articles, 16 articles were included in this review. (3) Results: Among the 16 studies that investigated the efficacy of treatment programs for managing chronic pain in patients with FHP, 11 investigated the effect of exercise programs, and 5 investigated the effect of manual therapy. Patients reported significant improvement in pain and disability after receiving treatment programs such as corrective postural exercises and special manual therapy techniques. (4) Conclusions: Various treatment programs, including postural corrective exercises and manual therapy, are beneficial for improving pain and disability in patients with FHP.

## 1. Introduction

Proper posture refers to a state of musculoskeletal balance that imposes a minimum amount of stress and strain on body structures [1]. Many people suffer from poor posture, and postural abnormalities can have a negative impact on musculoskeletal balance and psychological health [2]. A poor or altered posture of the spine can put considerable pressure on the surrounding muscles and ligaments, which can eventually affect the stability of the spine. It can also cause strain and fatigue on the surrounding structures, which often leads to pain [3].

Forward head posture (FHP) is one of the most common cervical postural deviations and is characterized by head protrusion or forward head placement in relation to the shoulder in the sagittal plane [4]. Sustained FHP is associated with an imbalance in muscles around the cervical spine, such as the shortened (or tightened) upper trapezius (TPZ), sternocleidomastoid (SCM), levator scapulae, suboccipital muscles, and lengthened (or weakened) deep neck flexor muscles [5].

FHP is associated with various disorders, such as trigger points in the suboccipital muscles, temporomandibular disorder, tension-type headache, and dyskinesia [5]. Additionally, previous systematic reviews have demonstrated that a significant relationship exists between FHP and neck pain and that FHP increases the risk of neck pain [6,7]. Therefore, correcting or minimizing FHP may be helpful in the management of neck pain.

Various treatment programs such as exercises, manual therapy, and modalities have been applied to treat neck pain and disability in patients with FHP [8]. However, to the best of our knowledge, no review has investigated the efficacy of these treatment programs. Therefore, the aim of this study was to investigate whether treatment programs are effective in the management of neck pain in patients with FHP.

## 2. Materials and Methods

**Figure 1:**
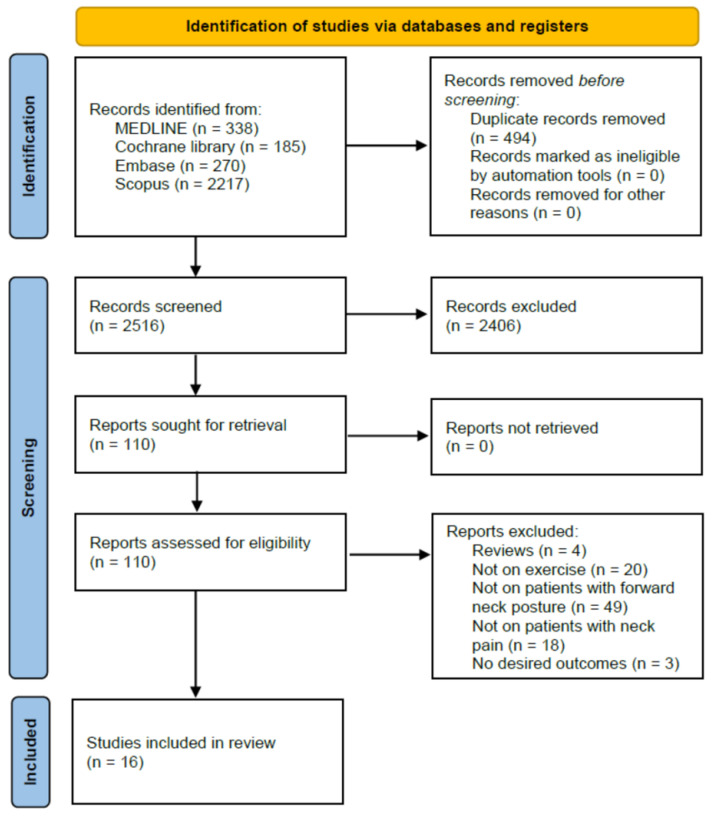
Flow diagram of the study selection process.

The protocol was registered on the international platform of registered systematic reviews and meta-analysis protocols (registration number: INPLASY202390061). A MEDLINE (PubMed), Embase, Cochrane Library, and Scopus databases search was conducted for English language articles on patients with chronic neck pain and FHP, published until 12 April 2023. To identify potentially relevant articles, the following key search phrases were combined: ‘forward head posture’ and ‘pain’. The following inclusion criteria were applied for the selection of articles: (1) patients with chronic pain, (2) patients diagnosed with FHP, (3) human studies, and (4) studies written in English. Relevant studies were selected according to the flow diagram shown in Figure 1. In total, 5516 potentially relevant articles were identified. After reading the titles and abstracts and assessing their eligibility based on full-text articles, 16 articles were included in this review.

## 3. Results

Sixteen studies investigated the efficacy of treatment programs in managing chronic pain in patients with FHP. All studies were randomized controlled trials (RCTs) that enrolled patients with FHP who had chronic neck pain or discomfort, mostly for >3 months. The patients were diagnosed with FHP, which is commonly defined as a craniovertebral angle (CVA) of <50° (diagnostic range < 44–53°). Studies have evaluated the efficacy of treatment programs using outcome measures such as pain scales (visual analog scale (VAS) and numerical rating scale (NRS)), neck disability index (NDI), and CVA.

Among the 16 studies included, 11 investigated the effect of exercise programs [6,9,10,11,12,13,14,15,16,17,18], and 5 investigated the effect of manual therapy [19,20,21,22,23] for the management of pain and disability in patients with FHP (Table 1).

### 3.1. The Effect of Exercise Programs

Eleven studies compared the effectiveness of exercise programs for managing pain and disability in patients with FHP [9,10,11,13,14,15,16,17,18]. In 2016, Im et al. directly compared the effect of scapular stabilization exercises (eight patients) with that of relaxation exercises (seven patients) [14]. Scapular stabilization exercises were performed in the supine, quadruped, and sitting positions with three laps of 10 repetitions, and the details of the relaxation exercises were not described further in this study. The two programs were performed three times/week for 4 weeks. The results showed that patients who performed scapular stabilization exercises showed greater improvements in CVA, VAS, NDI, and quality of life scores than those who performed relaxation exercises. In 2019, Fathollahnejad et al. enrolled 60 patients with forward head and rounded shoulder postures to investigate the effects of stabilization exercises [13]. Stabilization exercises included strengthening exercises for the periscapular muscles (Y to W, L to W, and scapular protraction). Patients who received stabilization exercises (40 patients) three times a day for 6 weeks showed pain reduction and improvement in function and posture compared to patients who were educated with postural correction on daily activities (home exercises) three times/week. These studies emphasize the importance of normalizing muscular activity to improve posture and pain in patients with FHP.

Sikka et al. (2020) enrolled 30 adolescents with chronic neck pain, FHP, and neck disability (NDI < 24) who use computers regularly [17]. Patients received either deep cervical flexor (DCF) training and postural education (15 patients) or postural education only (15 patients) for four sessions per week for 4 weeks. Adding DCF training to postural education improved neck pain and functional status; however, the effect was not significantly greater than that of postural education alone.

In 2021, Kang et al. investigated the effects of a combination of scapular stabilization and thoracic extension exercises [15]. This program included scapular retraction/mobilization/dynamic stabilization exercises and was compared with cervical stabilization exercises (16 vs. 16 patients). The exercises were performed three times/week for 6 weeks. The results of this study showed that both scapular and cervical stabilization exercises were effective in improving CVA, pain, and disability but did not explain which program was more effective. In the same year, Abadiyan et al. reported that the group that had an added smartphone app plus global postural re-education (GPR, including anterior and posterior muscle chain stretching exercises) of four sessions/week for 8 weeks showed better outcomes than the GPR alone and control (ergonomic education only) groups (20 vs. 20 vs. 20 patients) [9]. The GPR with a smartphone application showed better results in improving pain, FHP, and endurance than the GPR alone and control groups. It has also been suggested that using smartphone applications could be beneficial in reducing the rate of medical errors and the time required to care for a patient [24].

The effect of exercise programs on the management of chronic pain in patients with FHP has been continuously reported in other studies. In 2022, Dareh-Deh et al. conducted an RCT to investigate the effectiveness of exercise in patients with FHP [12]. A total of 60 smartphone users with non-specific chronic neck pain and FHP were divided into a treatment group (therapeutic exercises with or without respiratory exercises, 40 patients) or a control (20 patients) group. The therapeutic exercises included resistance and stretching exercises (e.g., side-lying external rotation, prone horizontal abduction with external rotation, and static levator scapulae stretch) performed three times/week for 8 weeks. The treatment group showed an observable decrease in pain compared to the control group, which reported no significant reduction in pain. This study suggests that exercise improves the balance between cervical muscle activity and posture, which may lead to pain reduction. Mohamed et al. (2022) investigated the effects of exercise on pain and disability [6]. The study enrolled 70 patients with cervical radiculopathy (pain radiating to one or both arms) or FHP (CVA of < 50°). Patients who received biofeedback FHP corrective exercises for 8 weeks showed significant improvements in CVA, referred arm pain, and NDI after 4 and 8 weeks compared to patients in the control group who did not receive any treatment. These two studies showed that when compared with controls who did not receive any treatment, therapeutic exercises were effective for correcting postural deviations and improving pain and disability in patients with FHP.

The effect of cervical stabilization exercises on chronic neck pain in patients with FHP was also reported by Arif et al. in 2022 [11]. Twenty patients underwent cervical stabilization exercises with conventional treatment (heating pad, transcutaneous electrical nerve stimulation (TENS), and cervical isometric exercises) three times/week for 4 weeks, and twenty patients received conventional treatment only. The results showed that performing cervical stabilization exercises (pillows placed beneath the cervical spine in the supine position with the patient nodding the head in cervical flexion) with isometric exercises was more effective than conventional treatment in reducing CVA, pain, and neck disability.

An RCT by Abd El-Azeim et al. also reported that adding scapular stabilization exercises to corrective postural exercises (30 patients) increased the CVA and pressure pain threshold and decreased disability compared with corrective postural exercises only (30 patients) [10]. The exercises were performed three times/week for 10 weeks. This study emphasized that scapular exercises helped relocate the abnormally aligned scapula, which induced improvement in neck muscle activities. Korsrokiani et al. (2022) addressed the importance of adding lumbar motor control training to DCF motor control training by demonstrating that this program improved pain and disability in patients with FHP [16]. Compared to patients in the control group (38 patients) who were only educated with postural corrections and heat/cold treatment, patients who received this combination treatment (38 patients) three times/week for 8 weeks showed better scores for pain, disability, DCF endurance, and health status. These two studies showed that combining scapular stabilization and lumbar motor control training was helpful in relieving pain and disability in patients with FHP.

Most recently, in 2023, Suwai et al. compared the effect of cervical traction orthotics (DennerollTM) and mirror image exercises (treatment group, 33 patients) with standardized exercises, which included stretching and strengthening exercises of the cervical muscles (control group, 33 patients) [18]. Treatment programs were administered to 66 patients with chronic neck discomfort and FHP over 18 sessions (3 sessions/week for 6 weeks). The treatment group showed greater immediate improvement in outcomes than the control group, and this effect was maintained even at the 3-month follow-up. Sustained extension loading provided via the cervical traction orthotic device helps restore cervical lordosis and improve anterior head translation of the FHP by stretching the discs, ligaments, and muscles of the cervical spine [25].

### 3.2. The Effect of Manual Therapy

Five studies investigated the effects of manual therapy with or without exercise [19,20,21,22,23]. In 2017, Cho et al. conducted an RCT to investigate the effect of a combination of joint mobilization and therapeutic exercises in improving pain [19]. Thirty-two patients underwent either upper thoracic spine mobilization and mobility exercises with high-intensity exercises against gravity (thoracic group, sixteen patients) or upper cervical spine mobilization and stabilization exercises with low-intensity isometric exercises (cervical group, sixteen patients). The results showed that the thoracic group showed significant improvements in CVA, NRS, and NDI after 10 sessions compared to the cervical group. Two years later, another RCT was conducted to compare the efficacy of upper cervical and thoracic spine mobilization (15 patients) and DCF exercises (16 patients) [20]. Patients who received ten sessions of upper cervical and thoracic spine mobilization showed better overall short-term outcomes in terms of CVA, NRS, and respiratory function than those who underwent a cervical spine stabilization (DCF) exercise program. This study emphasizes that enhancing the movement of the cervical and thoracic spine via joint mobilization is beneficial for pain and disability in patients with FHP.

The effects of manual therapy were also reported by Kim et al. in 2021 [22]. Joint mobilization treatments to the cervicothoracic junction (C7-T3) (11 patients) and upper cervical spine (C0-C1) (11 patients) were administered for 12 sessions over 4 weeks, and both treatments were beneficial for improving pain, dysfunction, and muscle activity in patients with FHP. Interestingly, joint mobilization to the cervicothoracic junction resulted in a greater improvement in CVA and ROM of the cervical extension than that of the upper cervical spine. Manual therapy was recommended in this study, as it was effective in improving cervical mobility and posture.

In the same year, Mylonas et al. compared the effects of instrument-assisted soft tissue mobilization (IASTM) (10 patients) and classical massage (10 patients) [23]. The IASTM technique is aimed at the myofascial release of shortened structures around the cervical and thoracic spines. These treatment programs were performed with neuromuscular exercises three times/week for 4 weeks. Although pain intensity decreased in both groups, the IASTM technique and neuromuscular exercises resulted in greater improvements in CVA, ROM of cervical flexion and extension, and NDI than simple massage with neuromuscular exercises.

The combined effect of the muscle energy technique (MET) and postural correction exercises was reported in 2022 by Joshi et al. [21], who enrolled 48 patients with chronic neck pain and FHP. Patients received a combination of MET and postural correction exercises (chin tuck and pectoral stretch) three times/week for 3 weeks, and they were compared with controls who only received strengthening exercises (DCF, rhomboids, lower TPZ, and serratus anterior) and stretching (pectoralis) exercises. The results showed that the combination of MET and exercise resulted in significantly greater decreases in CVA, pain, and disability compared to controls.

## 4. Discussion

To the best of our knowledge, this is the first review to summarize the effectiveness of neck pain treatment programs in patients with FHP. Postural deviation, such as FHP, increases the mechanical loads on the structures around the cervical spine, which may cause pain and increase the risk of degenerative changes and disc herniation. Neck pain associated with FHP is associated with poor posture, workload, and structural disorders that may affect activities of daily living, emotional control, and quality of life [26]. The therapeutic options for FHP with neck pain include exercise and manual therapy and modalities [8]. This review was the first to demonstrate that treatment programs such as exercises and manual therapy were beneficial for improving FHP (as measured via CVA), pain (as measured via VAS and NRS), and disability (as measured via NDI).

CVA is one of the common methods for the assessment of FHP. It is identified at the intersection of a line joining the midpoint of the tragus of the ear to the skin covering the C7 spinous process and a horizontal line passing through the C7 spinous process [27]. Smaller CVA represents more severe FHP, and studies included in this review enrolled patients with CVA < 50°.

**Figure 2:**
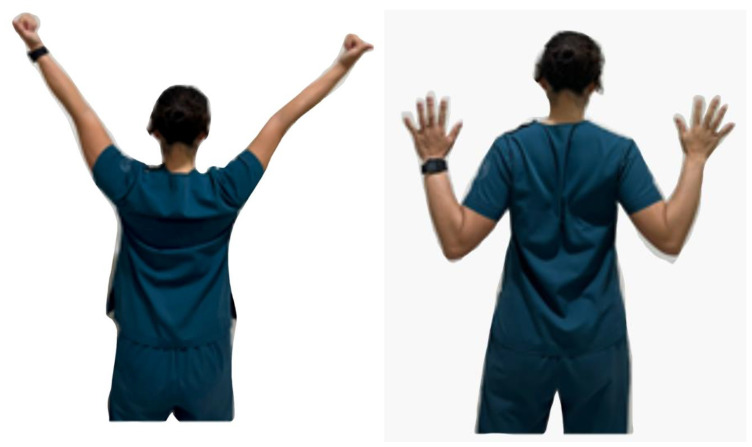
An example of a scapular stabilization exercise (Y to W).

**Figure 3:**
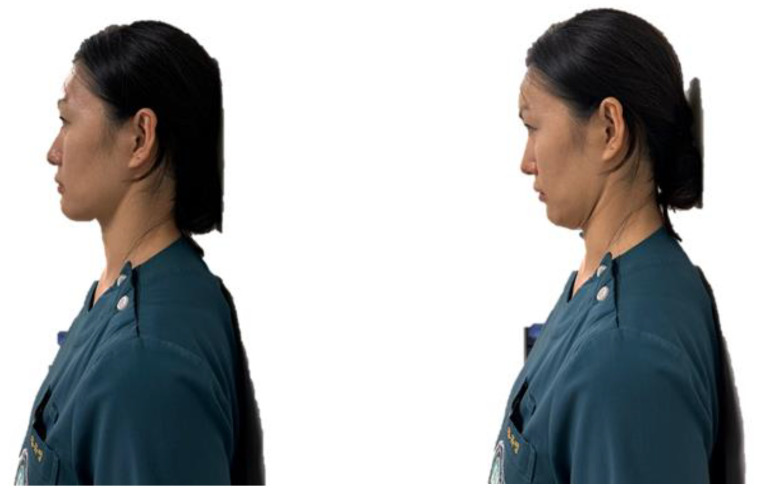
An example of a cervical stabilization exercise (chin tuck).

Common postural corrective exercise programs for FHP are cervical or scapular stabilization exercises, stretching and strengthening exercises (Figure 2 and Figure 3), proprioceptive training, sensorimotor training, and neuromuscular stabilization exercises [28]. Performing exercises helps decrease the strain and fatigue of the cervical muscles, which are caused by postural deviations of the cervical spine, and restores the stability of the structures. Postural corrective exercises may improve cervical spine alignment and alleviate muscle imbalance by decreasing cervical muscle activity in the upper trapezius, SCM, scalene, and cervical erector spinal muscles [10]. They can also inhibit superficial muscle spasms and fatigue. Relieving the overall tension caused by muscle balance can positively influence pain reduction and reduce disability in patients with chronic neck pain and FHP.

The mechanism underlying the positive effect of exercise on pain is that, as intrafusal fibers are reset with exercise, the cycle of muscle tension, impaired circulation with metabolite accumulation, and myofascial pain may be reduced [29]. Because exercise programs enhance between-muscle function, they can have beneficial effects on pain reduction. Functional balance between muscles is important; motor control training improves coordination between the DCF, superficial cervical flexors, and deep lumbar spinal muscles [16]. Additionally, strong isometric contractions of the neck muscles can activate muscle stretch receptors and trigger the release of beta-endorphins from the pituitary gland, which may lead to pain reduction [30].

Scapular stabilization exercises help relocate the abnormal position of the scapula, and proper alignment of the scapula induces structural changes and improves the muscle activities of the neck and back muscles [31]. Consequently, the adverse mechanical loads caused by awkward kinetics and positions of the scapula and cervical spine may be reduced. Therefore, scapular stabilization exercises also seem important for managing pain and disability in patients with FHP. Achieving a neutral cervical and craniocervical posture in patients with neck pain by performing stabilization exercises may induce effective load sharing within the muscle system. Proper load distribution in the musculoskeletal system can reduce focal end-range stress on the sensitized passive structures of the cervical spine [32].

Treatment programs that include manual therapy, with or without exercise, appear to be effective in managing neck pain and disabilities in patients with FHP. Manual therapies such as joint mobilization and joint manipulation are used to improve joint mobility and adhesion between soft tissues, increase ROM, and enhance the somatosensory system [33]. Manual therapy in patients with FHP can enhance flexion of the upper cervical spine and extension of the upper thoracic spine [19]. The mechanism underlying the effect of manual therapy is that the application of a continuous passive stimulus to soft tissues may change the sensitivity of mechanical receptors [34], helping reduce the mechanical stress of the pain generator and improving the biomechanical relationship between the cervical and thoracic spines [19].

Special manual therapy techniques are effective for treating neck pain in patients with FHP. Manual therapy can be divided into hands-on therapy techniques, such as soft tissue mobilization and massage, and techniques that use therapeutic equipment, such as IASTM. IASTM is a targeted soft tissue myofascial technique that uses special equipment to improve the mobility of connective tissue and myodynamic adaptations. This technique has been found to be helpful in reducing postural stress and improving neck pain and disability [23]. In addition, the MET combined with postural correction exercises decreased neck pain and disability in patients with FHP. MET is an active manual technique that involves a patient performing an isometric contraction and post-isometric relaxation of the muscles to improve joint mobility and increase ROM [35]. Applying the MET in combination with postural correction exercises helps increase muscle flexibility by inducing viscoelastic changes in the muscle and reducing muscle tension [21]. It helps restore normal blood and lymphatic circulation by altering the interstitial pressure, which may wash out the nociceptive stimulants of pain. Overall, it appears that a combination of manual therapy and therapeutic exercise effectively manages neck pain in patients with FHP.

Overall, correcting improper cervical, thoracic, and scapular kinematics and muscular imbalance via exercise and manual therapy seems beneficial for managing pain and disability in patients with FHP.

### Limitations

The first limitation is that the studies did not report long-term effects and are, therefore, unknown. Secondly, many studies had small sample sizes and were limited to patients in the chronic phase. Studies with manual therapy were relatively very few in comparison to stabilization exercises. In addition, the study groups were not matched for age or sex. Further studies should be conducted in different age groups to investigate the activity of specific muscles in patients with neck pain and FHP.

## 5. Conclusions

Treatment programs seem effective in managing neck pain and disability in patients with FHP. Various treatment programs, including postural corrective exercises and manual therapy, seem beneficial for improving pain and functional status in these patients. Thus, treatment programs are recommended to improve FHP, pain, and disability in these patients.

## Figures and Tables

**Table 1:** Characteristics of included studies.

#	First Author	Year	Study Design	Treatment Group	Control Group	No. of Patient (Active/Control)	Duration of Intervention	Outcome Parameters	Results
1	Im et al. [14]	2016	RCT	Scapular stabilization exercises	Relaxation exercises	15 (8/7)	3 sessions, 4 weeks (total 12 sessions)	CVA, upper trapezius/serratus anterior muscle acitivity, NDI, VAS, quality of life	Patients with scapular stabilization exercises showed greater improvement in CVA, VAS, NDI, and quality of life scores than the controls.
2	Cho et al. [19]	2017	RCT	Upper cervical mobilization+ stabilization exercise	Upper thoracic mobliziation + mobility exercise	32 (16/16)	4 weeks (total 10 sessions)	CVA, cervical ROM, NRS, NDI, GRC	The thoracic group showed significant improvements in CVA, NRS, and NDI compared to the cervical group.
3	Cho et al. [20]	2019	RCT	Mobilization	Exercise	31 (15/16)	4 weeks (total 10 sessions)	CVA, NRS, respiratory function, GRC	Patients who received the mobilization program showed better overall short-term outcomes in CVA, NRS, and respiratory function compared to the patients who received the exercise program.
4	Fathollahnejad et al. [13]	2019	RCT	Stabilizating exercises and manual therapy	Stabilizating exercises only vs. home exercises	60 (20/20/20)	3 sessions, 8 weeks (total 18 sessions)	VAS, PILE test, photogrammetry (FHA and FSA)	Patients who received stabilization exercises with or without manual therapy showed pain reduction and improvement in function and posture compared to patients who performed only home exercises.
5	Sikka et al. [17]	2020	RCT	Deep cervical flexor training and postural education	Postural education	30 (15/15)	4 sessions, 4 weeks (total 12 sessions)	CVA, VAS, NDI	Adding DCF training and postural education was beneficial for improving neck pain and functional status, but it was not significantly superior to postural education alone.
6	Abadiyan et al. [9]	2021	RCT	Global postural re-education +smartphone app	Global postural re-education only vs. control (neck education and exercise)	60 (20/20/20)	4 sessions, 8 weeks (total 32 sessions)	VAS, NDI, SF-36, photogrammetry, PILE test	Adding a smartphone app to an 8-week global postural re-education (GRP) showed better results in improving pain, FHP, and endurance compared to the GPR alone and the controls.
7	Kang et al. [15]	2021	RCT	Scapular stabilization and thoracic extension exercises	Cervical stabilization and stretching exercises	32 (16/16)	3 sessions, 6 weeks (total 18 sessions)	CVA, respiratory pressure, respiratory function, VAS, NDI	Both treatment programs were effective in improving CVA, pain, and disability.
8	Kim et al. [22]	2021	RCT	Joint mobilization and motor control training in upper cervical spine (C0-C1) and cervicothoracic junction (C7-T3)	Joint mobilization and motor control training only in upper cervical spine	22 (11/11)	3 sessions, 4 weeks (total 12 sessions)	CVA, NRS, NDI, ROM, muscle activity	Both groups showed beneficial effects in pain, dysfunction, and muscle activity after the treatment.
9	Mylonas et al. [23]	2021	RCT	Instrument-assisted soft tissue mobilization (IASTM) with neuromuscular exercises	Classical massage and neuromuscular exercises	20 (10/10)	2 sessions, 4 weeks (total 8 sessions)	CVA, VAS, NDI ROM, strength	IASTM technique and neuromuscular exercises resulted in greater improvement in CVA, ROM of cervical flexion and extension, and NDI than a simple massage with neuromuscular exercises.
10	Arif et al. [11]	2022	RCT	Cervical stabilization exercises with conventional treatment (heating pad, TENS, and cervical isometric exercises)	Conventional treatment	40 (20/20)	3 sessions, 4 weeks (total 12 sessions)	CVA, NRS, NDI, single breath count, and spirometry	Performing cervical stabilization exercises with isometric exercises was more effective in reducing CVA, pain, and neck disability than the conventional treatment.
11	Dareh-Deh et al. [12]	2022	RCT	Routine therapeutic program with respiratory exercises	Routine therapeutic program vs. control	60 (20/20/20)	3 sessions, 8 weeks (total 24 sessions)	FHA, VAS, activity of specific muscles, and respiratory patterns	Both experimental groups showed an observable decrease in pain following the interventions compared to the control group.
12	Abd El-Azeim et al. [10]	2022	RCT	Adding scapular stabilization to postural corrective exercises	Only postural corrective exercises	60 (30/30)	3 sessions, 10 weeks (total 30 sessions)	CVA, pressure pain threshold, Arabic NDI, cervical flexor, and extensor muscle endurance	Adding scapular stabilization exercises to postural corrective exercises increased CVA and pressure pain threshold and decreased disability compared to postural corrective exercises only.
13	Joshi et al. [21]	2022	RCT	MET+ postural correction exercises	Strengthening and stretching exercises	48 (23/25)	3 sessions, 3 weeks (total 9 sessions)	CVA, NRS, NDI	Patients who received MET and posture correction exercises showed significantly greater decreases in CVA, pain, and disability compared to the controls.
14	Khosrokiani et al. [16]	2022	RCT	Lumbar motor control exercises +DCF motor control training	DNF motor control training alone vs. postural corrections and heat/cold treatments	113 (38/37/38)	3 sessions, 8 weeks (total 24 sessions)	VAS, NDI, deep cervical flexor muscular endurance, and health status	The addition of lumbar motor control training enhances the effectiveness of deep cervical flexor motor control training on neck pain, neck disability, and deep cervical flexor endurance.
15	Mohamed et al. [6]	2022	RCT	Biofeedback posture corrective exercises	No treatment	70 (35/35)	3 sessions, 8 weeks (total 24 sessions)	CVA, NRS, NDI, biofeedback on reaction time, and central somatosensory conduction time	Patients in the study group showed significant improvement in CVA, referred arm pain, and NDI compared to the patients in the control group.
16	Suwadi et al. [18]	2023	RCT	Cervical traction orthotic and mirror image exercises	Standardized exercises	66 (33/33)	3 sessions, 6 weeks (total 18 sessions)	CVA, VAS, Berg balance score, head repositioning accuracy, cervical ROM	The treatment group showed greater immediate improved outcomes, and this effect was maintained even at the 3-month follow up compared with the control group.

CVA, craniovertebral angle; DCF, deep cervical flexor; EMG, electromyography; FHA, forward head angle; GRC, global rating of change; NDI, neck disability index; NA, not applicable; NRS, numeric rating scale; MET, muscle energy technique; MT, middle trapezius; NDI, neck disability index; PILE, progressive isoinertial lifting evaluation; PPT, pressure pain threshold; RCT, randomized controlled trial; ROM, range of motion; SSA, sagittal shoulder angle; TENS, transcutaneous electrical nerve stimulation; VAS, visual analog scale.

## Data Availability

No new data were created or analyzed in this study. Data sharing is not applicable to this article.
